# Randomly positioned gold nanoparticles as fluorescence enhancers in apta-immunosensor for malaria test

**DOI:** 10.1007/s00604-021-04746-9

**Published:** 2021-02-16

**Authors:** Antonio Minopoli, Bartolomeo Della Ventura, Raffaele Campanile, Julian A. Tanner, Andreas Offenhäusser, Dirk Mayer, Raffaele Velotta

**Affiliations:** 1 Institute of Biological Information Processing (IBI-3), Bioelectronics, Forschungszentrum Jülich, 52425 Jülich, Germany; 2 Department of Physics “E. Pancini”, University of Naples “Federico II”, Via Cintia 26, 80126 Naples, Italy; 3 School of Biomedical Sciences, University of Hong Kong, Hong Kong, SAR China

**Keywords:** Nanoplasmonics, Plasmon-enhanced fluorescence, Photochemical immobilization technique, Antibody-aptamer biosensor, Malaria marker, Gold nanoparticle array

## Abstract

A plasmon-enhanced fluorescence-based antibody-aptamer biosensor — consisting of gold nanoparticles randomly immobilized onto a glass substrate via electrostatic self-assembly — is described for specific detection of proteins in whole blood. Analyte recognition is realized through a sandwich scheme with a capture bioreceptor layer of antibodies — covalently immobilized onto the gold nanoparticle surface in upright orientation and close-packed configuration by photochemical immobilization technique (PIT) — and a top bioreceptor layer of fluorescently labelled aptamers. Such a sandwich configuration warrants not only extremely high specificity, but also an ideal fluorophore-nanostructure distance (approximately 10–15 nm) for achieving strong fluorescence amplification. For a specific application, we tested the biosensor performance in a case study for the detection of malaria-related marker *Plasmodium falciparum* lactate dehydrogenase (*Pf*LDH). The proposed biosensor can specifically detect *Pf*LDH in spiked whole blood down to 10 pM (0.3 ng/mL) without any sample pretreatment. The combination of simple and scalable fabrication, potentially high-throughput analysis, and excellent sensing performance provides a new approach to biosensing with significant advantages compared to conventional fluorescence immunoassays.

**Supplementary Information:**

The online version contains supplementary material available at 10.1007/s00604-021-04746-9.

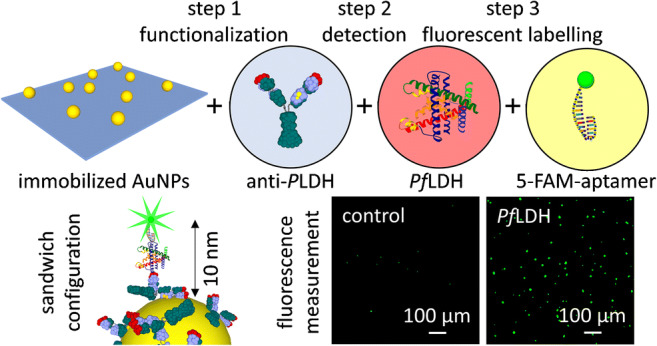

## Introduction

Fluorescence-based techniques are widely employed and rapidly emerging as a leading methodology in biotechnology, biomedicine, and life sciences [1, 2]. High fluorophore brightness is crucial when remarkable sensitivity is required, particularly in presence of interfering background arising from biological systems or complex matrices. Several efforts have been carried out to amplify the fluorescence signal, including fluorescence enhancement (FE) provided by plasmonic nanostructures [3, 4]. Although the mechanism behind the plasmon-enhanced fluorescence (PEF) is still not entirely understood [5], FE results from the resonant coupling between the plasmon of the metal nanostructure and the nearby fluorophore that can lead to both excitation and emission amplification [6]. The possibility to adapt nanostructured platforms to the conventional fluoroassays as well as to go beyond the enzyme-linked immunosorbent assay (ELISA) is still the main long-standing goals of PEF-based techniques [5].

Plenty of plasmonic substrates consisting of two-dimensional (2D) arrays of metal nanostructures have been developed as PEF-based biosensing platforms [5, 7–9]. In the last few years, several milestones have been achieved in terms of FE factor (up to 10^5^-fold) [10] and limit of detection (LOD) (down to fM level) [9]. However, the complexity of these approaches usually limits the success of PEF-based fluoroassays in point-of-care testing and mass screening [3]. 2D arrays of gold nanoparticles (AuNPs) are attractive candidates as fluorescence enhancers because of cost-effective and scalable fabrication, tunable plasmonic properties, and wide substrate versatility [11]. In a previous paper, we demonstrated the effectiveness of PEF-based apta-immunosensor—relying on ordered arrays of AuNPs—for the detection of malaria biomarker at the femtomolar level in whole blood [12]. Although ultrasensitive detection is highly desirable in many assays, there is still a variety of applications in which the need for an extremely low LOD can be loosened in favour of a more cost-effective fabrication procedure. Thus, we have realized a different fluorescence enhancer consisting of AuNPs randomly immobilized onto a glass substrate through silane surface modification [11]. Despite the lack of order in the AuNP arrangement entailed a reduction of the fluorescence amplification, the LOD provided by this biosensor in a complex matrix like human blood lies in the picomolar range. This was demonstrated by detecting *Plasmodium falciparum* lactate dehydrogenase (*Pf*LDH) in spiked human blood without any pretreatment.

Malaria-infected individuals exhibit *Pf*LDH concentrations at the nanomolar level in red blood cells with picomolar levels in serum [13]; very low concentrations (down to femtomolar level) are also recently detected in malaria patient saliva [14]. Gold standard methods rely on either parasite microscope observation [15] or polymerase chain reaction (PCR) [16]. Both methods are time-consuming and require well-equipped laboratories as well as skilled personnel; thus, simple devices as biosensors are sought for point-of-care testing and mass screening. Conventional rapid diagnostic tests (RDTs) for malaria diagnosis based on lateral flow assay offer fast and cost-effective *Pf*LDH detection in pretreated blood. In these tests, red blood cells are lysed both to increase the *Pf*LDH concentration and to facilitate blood flow along the dipstick. However, such malaria RDTs are not appropriate for early diagnosis and mass screening because of their poor LOD and difficulties of pre-functionalized device transportation and storage in a tropical environment [17, 18].

Other colorimetric approaches have been explored, but the impossibility to carry out the measurement directly in blood or whole serum strongly limits the practical application of these biosensors [19–21]. In this regard, ELISA offers an established colorimetric strategy to reach a remarkable LOD in lysed blood or whole serum, albeit suffering from both the high cost of the kits and the time-consuming assay [22]. The aptamer-tethered enzyme capture (APTEC) approach represents a promising method to reduce the high cost of the antibody-based assays [23, 24]. Nevertheless, still some challenges in the LOD and the requirement of complex microfluidic systems strongly limit their practicality. Very low LODs are achieved by electrochemical biosensors, but they only work with serum [25, 26] or serial dilutions are needed if a quantitative measurement is required [27]. Fluorescence-based biosensors show a great potential for point-of-care applications since the sensitivity can be drastically increased by using plasmon nanostructures as a fluorescence enhancer and the sample pretreatment can be avoided [12]. However, the turbidity of the whole blood could be detrimental for such techniques; thus, a pretreatment or serum extraction might be needed [28, 29].

The PEF-based assay described here combines the plasmonic features of AuNPs with a unique photochemical functionalization technique giving rise to a strong fluorescence amplification and high sensitivity. The photochemical immobilization technique (PIT) offers a fast and simple strategy (only a UV lamp is required) to covalently tether Abs onto gold surfaces in such a way that one fragment antigen-binding (Fab) is exposed to the surrounding environment [30, 31]. The detection is carried out with a sandwich configuration in which Abs act as capture bioreceptors, whereas fluorescently labelled aptamers (Apts*) bind *Pf*LDH on the top thereby warranting extremely high specificity.

## Experimental

### Reagents and materials

Full details are displayed in Section 1 in ESM.

### Fabrication of randomly positioned AuNP array

Citrate-stabilized AuNPs were synthesized by chemical reduction of gold(III) chloride trihydrate with sodium citrate [32] (more details on the synthesis protocol are described in Section 2 in ESM).

Silanization process by (3-aminopropyl)triethoxysilane (APTES) was employed to chemically modify the substrate surface—consisting of 10 × 8 mm^2^ glass coverslips—allowing the random immobilization of citrate-stabilized AuNPs by simple adsorption via electrostatic interactions [11]. The procedure included five steps that are schematically shown in Section 3 in ESM.

The substrate was optically and morphologically characterized to retrieve information concerning the plasmon resonance wavelength and the shape and size of nanoparticles as well as the interparticle distance, respectively (technical details about the SEM image processing are reported in Section 4 in ESM).

A finite-difference time-domain (FDTD) method was adopted to simulate the optical response of the fabricated 2D AuNP array (technical details on the numerical simulation are described in Section 5 in ESM).

### Substrate biofunctionalization and blocking

Gold nanoparticle functionalization with pan malaria antibodies (anti-*P*LDH) was achieved by PIT. A volume of 1 mL aqueous solution of anti-*P*LDH (50 μg/mL) was irradiated by Trylight UV lamp for 30 s and flowed onto the substrate (more details on the PIT are reported in Section 6 in ESM). The functionalization and blocking were carried out by using a microfluidic circuit (technical details are described in Section 7 in ESM).

### Ab-*Pf*LDH-Apt* sandwich scheme realization

The desired amount of *Pf*LDH protein was spiked into 1 mL of uninfected whole blood to obtain the analyte concentration of interest. Blood samples were taken from the donators by means of a monovette tube containing ethylenediaminetetraacetic acid (EDTA) to prevent coagulation. Moreover, we had to dilute the blood to reduce the solution turbidity. A dilution of 1:100 in 1 mL of 25 mM Tris buffer was used throughout the experiment as good tradeoff for retrieving high signal as well as treatable solution.

The functionalized substrate was incubated with 1 mL of contaminated blood and the system was gently shaken for 2 h by a tilting laboratory shaker to improve the analyte diffusion and hence the probability to be captured by immobilized Abs (the incubation time could be reduced to 50 min in view of the results obtained in the binding kinetic study). Then, the sample was abundantly rinsed by Tris buffer (25 mM) and ultrapure water to remove blood residues and unbound proteins.

Thus, the substrate was transferred into 1 mL of PBS solution (10 mM) containing 0.1 μM of malaria Apts*. The system was gently shaken for 2 h in dark condition so that the Ab-*Pf*LDH-Apt* sandwich was realized (the incubation time could be reduced to 30 min in view of the results obtained in the binding kinetic study). Afterwards, the sample was copiously rinsed by PBS (10 mM) and ultrapure water to remove unbound Apts*.

### Analysis of the fluorescence images

In order to carry out a robust analysis, the fluorescence intensity corresponding to a single measurement was estimated by averaging the signals delivered by ten images randomly recorded on the substrate (full details on the fluorescence image acquisition and processing are reported in Sections 8 and 9 in ESM, respectively). Moreover, each substrate was used for a single *Pf*LDH concentration—rather than investigating additive concentrations on the same substrate—to inherently test the reproducibility of the detection procedure.

### Fluorescence detection process of *Pf*LDH in human blood

A detailed protocol on how to perform the measurement of *Pf*LDH concentration in human blood is provided in Section 10 in ESM.

## Results and discussion

### Operating principle of the PEF-based assay

**Fig. 1:**
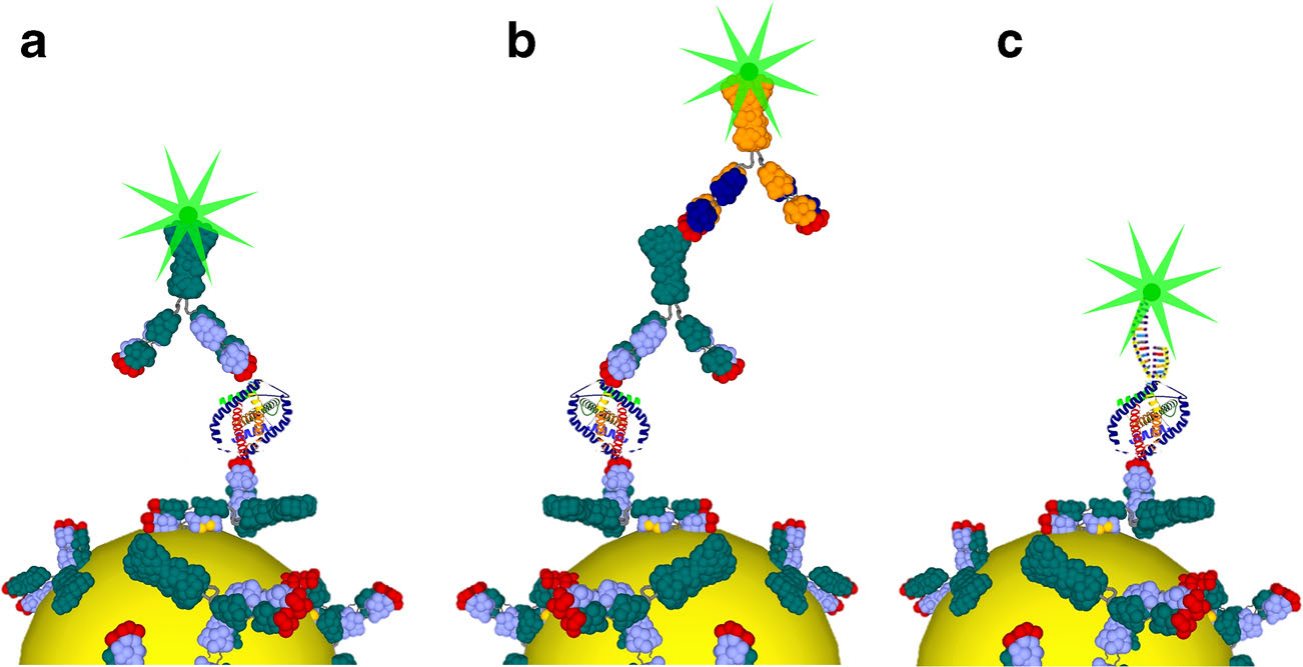
Possible detection schemes of a PEF-based biosensor with the bottom bioreceptor layer realized by Abs immobilized through PIT. Top bioreceptors can consist of monolayer of fluorescently labelled Abs (Abs*) (**a**), double layer of Abs-Abs* (**b**), or monolayer of Apts* (**c**)

Figure 1 shows the operating principle of the proposed PEF-based biosensor for three feasible configurations. Antibodies offer considerable advantages as a bottom bioreceptor layer in terms of surface biofunctionalization. The top fluorescently labelled bioreceptor layer can be conveniently adapted to the actual case study by simply switching among Abs and Apts. It is worth to underline that the combination of Abs and Apts* (Fig. 1c) provides a powerful approach not only to significantly improve the specificity [33] but also to enable optimal fluorophore-nanostructure distance (approximately 10–15 nm) for PEF-based sensors.

The Ab-analyte-Apt* sandwich used in this work consisted of anti-*P*LDH as the capture bioreceptor layer that offers effective detection of any malaria biomarkers *Plasmodium* lactate dehydrogenase (*P*LDH) [34], whereas malaria Apts* used as the top bioreceptor layer warrants a cost-effective and highly specific targeting of *Pf*LDH with discrimination from *Plasmodium vivax* LDH (*Pv*LDH) [35].

### Morphological characterization of the 2D AuNP array

The colloidal solution of AuNPs was characterized by UV-Vis spectroscopy and scanning transmission electron microscopy (STEM) (see Section 11 in ESM). The extinction spectrum exhibits a narrow LSPR peak at 526.4 nm, as expected for 30-nm-diameter gold nanospheres in water [36]. STEM images show regular spherical monodisperse nanoparticles of 33 ± 5 nm diameter finding out excellent agreement with the optical characterization. Such AuNPs were used to fabricate two-dimensional array of randomly positioned gold nanospheres.

**Fig. 2:**
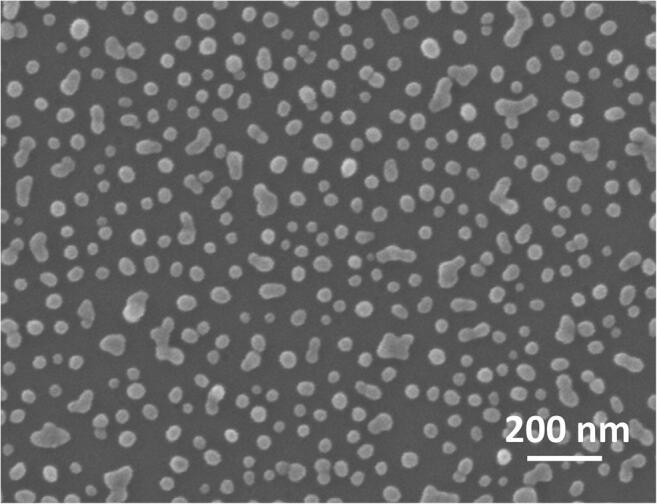
Scanning electron micrograph of the 2D AuNP array

The substrates were morphologically characterized by scanning electron microscopy (SEM). SEM micrographs show spherical nanoparticles of approximately 30-nm diameter randomly distributed onto the substrate as both single nanoparticles and clusters (Fig. 2). The presence of clusters is ascribable to the occurring of nanoparticle aggregation during the incubation of silane-modified substrates with citrate-stabilized AuNPs due to silane multilayer spots that could arise from the silanization process. Aiming at increasing the number of binding sites available for Ab-*Pf*LDH-Apt* complexes, the densest packing of nanoparticles was promoted during the substrate fabrication corresponding to 340 ± 30 AuNPs per μm^2^ (more details on the morphological characterization of the substrate are displayed in Section 12 in ESM).

### Optical response of the substrate

**Fig. 3:**
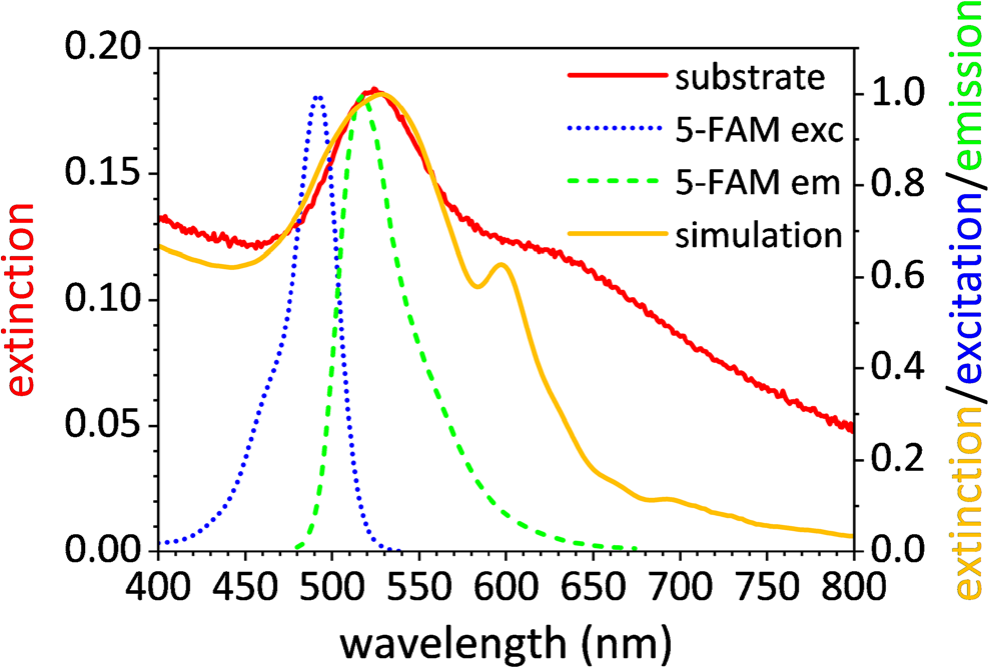
Experimental (red continuous line) and simulated (gold continuous line) extinction spectrum of the substrate; excitation (dotted blue line) and emission (dashed green line) spectrum of the fluorophore

The substrate was also optically characterized through UV-Vis spectroscopy by measuring its extinction spectrum. Figure 3 shows the experimental extinction spectrum of the bare substrate (red continuous line), which contains two plasmonic resonances occurring at (i) 524 nm and (ii) 620 nm. (i) Single nanoparticles far enough from their nearest neighbours (*d* > 3/2*D*) [37] contribute to the resonance occurring at smaller wavelength, as expected for 30-nm-diameter gold nanospheres in air [38], whereas (ii) AuNP clusters give rise to a shoulder at higher wavelength [38]. The coverage of AuNPs with a dielectric protein layer leads to a red-shift of the LSPR peak of 4 nm after the Ab-functionalization and any significant change in plasmon resonance after gold surface blocking as a result of the Ab close-packing arrangement offered by PIT (see Section 13 in ESM for more details on the functionalization study). The robustness of the proposed assay is also proved by the high reproducibility in the extinction spectrum of the bare substrates (Fig. S11a) as well as the LSPR red-shift due to the Ab-functionalization (Fig. S11b).

The superposition of the plasmonic resonance to the emission peak of 5-carboxyfluorescein (5-FAM) (green dashed line)—the fluorophore used in this experiment—provides an ideal condition for achieving a strong PEF amplification since the Ab-*Pf*LDH-Apt* sandwich warrants a mean fluorophore-nanoparticle distance of approximately 10 nm. The consistent agreement between the experimental extinction spectrum (red continuous line) and that worked out by numerical simulation (gold continuous line) provides assurance about the holding at the macroscopic level of the observed micrometric morphology.

### PEF-based antibody-aptamer assay performance

Firstly, the assay was kinetically characterized by measuring the fluorescence signal at a high *Pf*LDH concentration (1 μM) as a function of incubation time for both binding processes: (1) Ab-analyte and (2) Apt*-analyte. As it concerns the process (1), *Pf*LDH-spiked blood was preliminarily mixed with a solution containing malaria Apts* so that the resulting *Pf*LDH-Apt* complexes were fluorescently visible while the small size of Apts* did not significantly affect the diffusion and binding kinetics. Thus, functionalized substrates were incubated with *Pf*LDH-Apt* solution and fluorescence images were recorded at different incubation times (Fig. S12a). The process (2) was investigated by incubating the functionalized substrates with *Pf*LDH-spiked blood for a time long enough to warrant the reach of the dynamic equilibrium—as a consequence of the empirical study (1)—and then with the solution containing the malaria Apts* (Fig. S12b). Figure S13 shows the fluorescence intensity as a function of the incubation time for both the binding processes. The data are well fitted by the exponential curve

1 $$ F(t)={F}_{\mathrm{eq}}\left(1-{e}^{-\frac{t}{\tau }}\right) $$

where *F*_eq_ is the signal measured approaching the dynamic equilibrium while *τ* is the time constant of the binding process. Both the processes (1) and (2) exhibit similar kinetics with time constants of 50 ± 10 min and 30 ± 5 min, respectively. Thus, if a commercial fluorescence reader is used to record the signal, the whole analysis could be carried out within few hours, very attractive feature in terms of no time-consuming assay. The slight discrepancy in the asymptotic values *F*_eq_^(1)^ < *F*_eq_^(2)^ is ascribable to the less effective binding among the *Pf*LDH-Apt* complexes and the immobilized Abs since the Apts* might have targeted all analyte binding sites during their pre-incubation [39].

**Fig. 4:**
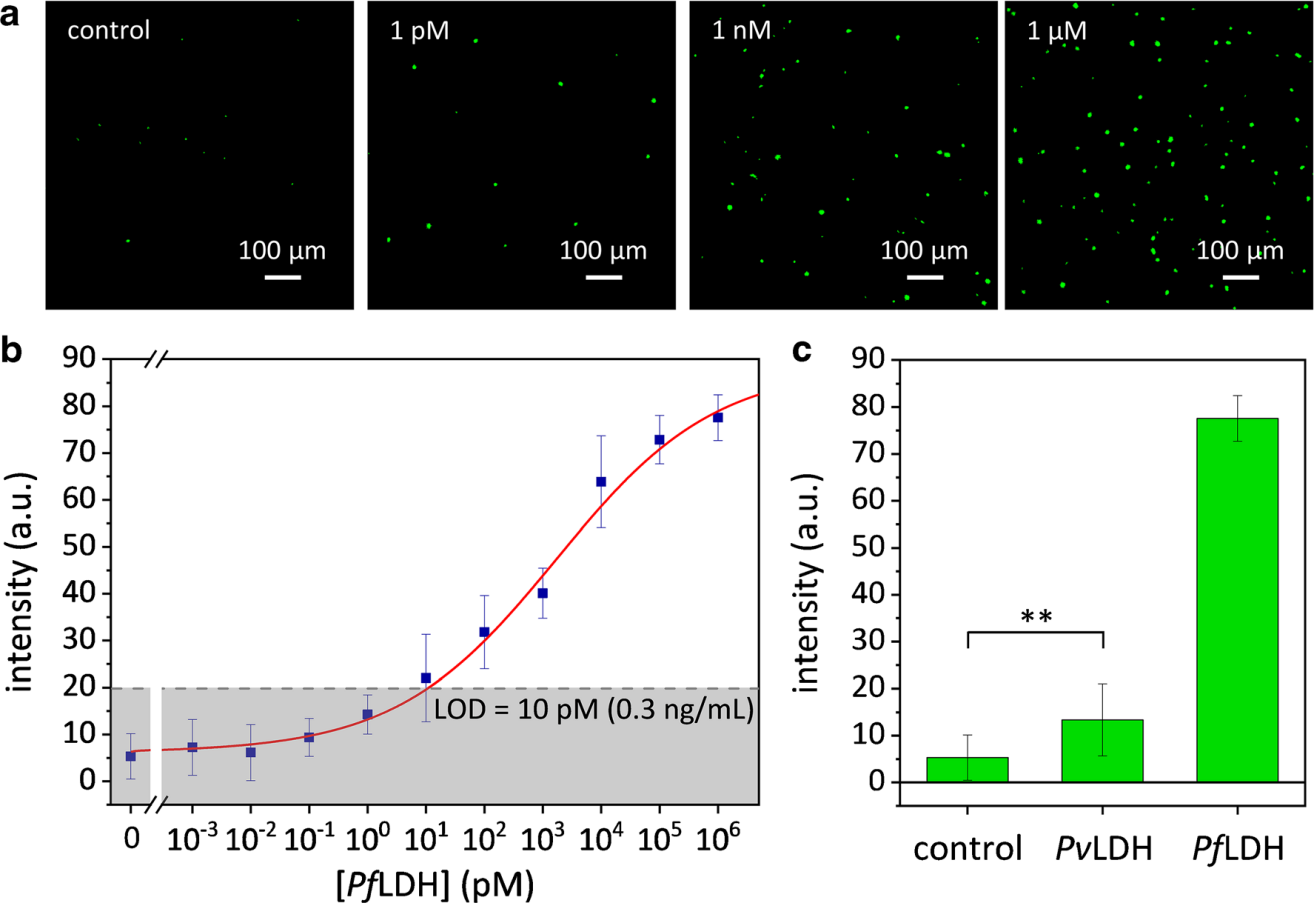
Fluorescence-based antibody-aptamer malaria assay. **a** Examples of processed fluorescence images recorded at different *Pf*LDH concentrations in spiked whole blood. **b** Calibration curve (fluorescence intensity vs *Pf*LDH concentration in whole blood) of the biosensor (LOD = 10 pM). **c** Specificity of the antibody-aptamer malaria assay against *Pv*LDH (**p value <0.001). The data are presented as mean value ± standard deviation and are representative of ten technical repeats

Afterwards, we explored a broad range of *Pf*LDH concentrations—from 1 fM to 1 μM referring to undiluted whole blood—that is of interest from a medical point of view [40]. Figure 4a shows some examples of fluorescence images recorded at different *Pf*LDH concentrations, in which the number of green spots is strikingly higher as compared to the control down to 1 pM (33 pg/mL) (see Fig. S14 for more images). The no zero fluorescence signal measured for the control arises from no specific binding of Apts* after uncontaminated blood was flowed onto the functionalized substrate possibly due to the presence of human LDH and other proteins in very high concentrations in real blood sample. Spot fluorescence is strongly related to spot area exhibiting linear correlation with similar behaviours regardless of analyte concentration (Fig. S15). The mean spot area turned out to be 34 μm^2^ corresponding to approximately 80 pixels.

Aiming at measuring the FE factor delivered by such a plasmonic substrate, 100 μL of a solution containing 250 fmol of Apts* was drop-casted onto a bare microscope slide in order to estimate the fluorescence *I*_0_ provided by the single fluorophore in free-space condition. We estimated the whole fluorescence intensity *F*_0_ by considering the dried drop area composed of a 12-mm-diameter circle surrounded by a 0.15-mm-thick annulus whose intensity was 10-fold higher than that measured in the inner region (Fig. S16). Inner region and annulus were separately sampled finding out that the contribution of the annulus to the whole intensity was minor, even though its intensity was locally much higher. Thus, the single fluorophore intensity could be retrieved by assuming *F*_0_ = *k*_ins_*N*_0_*I*_0_, where *N*_0_ is the number of Apts* (approximately 1.5 × 10^11^) and *k*_ins_ an instrumental constant depending on the acquisition parameters. On the other hand, in the presence of the plasmonic substrate, the fluorescence arose from separated emitters; hence, the whole intensity is given by *F*_PEF_ = *k*_ins_*N*_PEF_*I*_PEF_, where *N*_PEF_ is the number of fluorescence spots while *I*_PEF_ is their mean intensity. Since the FE factor is defined as *G* = *I*_PEF_/*I*_0_, our plasmonic substrate yielded a fluorescence amplification of 340.

Figure 4b shows the fluorescence intensity *F* as a function of *Pf*LDH concentration in whole blood. The data are well fitted by the four-parameter Hill equation [41].

2 $$ F\left(\left[ Pf\mathrm{LDH}\right]\right)={F}_1+\frac{F_2-{F}_1}{1+{\left(\frac{K}{\left[ Pf\mathrm{LDH}\right]}\right)}^n} $$

with *F*_1_ = 6 ± 2 arb. units, *F*_2_ = 88 ± 6 arb. units, *K* = (1.7 ± 0.9) × 10^3^ pM, the Hill coefficient *n* = 0.32 ± 0.06, and *χ*^2^ = 1.4. The detection range extends over 5 decades with a LOD of 10 pM (0.3 ng/mL)—estimated as three standard deviations above the control value (grey region in Fig. 4b).

To determine the specificity of the *Pf*LDH test in the PEF-based antibody-aptamer biosensor, we studied its response against the *Pv*LDH, which shares 90% residue identity with *Pf*LDH [42]. The *Pv*LDH was spiked into uninfected blood (1:100 diluted in 1 mL of 25 mM Tris buffer) to obtain a concentration of 10 nM (1 μM referring to undiluted whole blood). In Fig. 4c, fluorescence intensities provided by *Pf*LDH, *Pv*LDH, and control are compared revealing no significant cross-reaction detected with the main *Pf*LDH competitor. In fact, malaria Apts* used as the top bioreceptor layer warranted extremely high specificity only against *Pf*LDH protein [35], despite the Ab bottom bioreceptor layer might capture any *P*LDH malaria biomarkers.

Compared with existing malaria diagnostic test, our present apta-immunosensor is among the most sensitive optical-based sensors reported so far in combination with a broad dynamic range (DR) and extremely high specificity inherently ensured by the double biorecognition (Table 1). Moreover, no additional steps—such as red blood cell (RBC) lysis or serum extraction—are required to carry out the test. Although the measurement itself does not need complex equipment, an on-site laboratory is however required both to functionalize the substrate and to dilute the blood sample.

**Table 1:** An overview on recently reported nanomaterial-based methods for the detection of malaria

Transducer	Method	DR LOD	Matrix	Remarks	Ref.
Gold electrode	EC	80.3 pg/mL–3.5 μg/mL 26.9 pg/mL	Human serum	A centrifuge step is required to extract the human serum	[25]
Graphene oxide-glassy carbon electrode	EC	17–350 fg/mL 17 fg/mL	Lysed RBC solution	Extremely low LOD albeit the DR is below the relevant concentrations for the malaria diagnosis	[27]
Gold nanohole array	O-EC	35 pg/mL–35 μg/mL 49 pg/mL	Buffer	EC measurement is necessary to investigate low concentrations in DR	[26]
Colloidal gold nanoparticle	Col	Not specified 38 ng/mL	HEPES buffer	Centrifuge and lysis steps are required to carry out the measurement in real sample	[19]
Lateral flow immunoassay	Col	10–300 ng/mL 10 ng/mL	Buffer	Not suitable for early diagnosis Require a cold chain	[18]
Aptamer-tethered enzyme capture (APTEC)	Col	5 ng/mL–5 μg/mL 5 ng/mL	BSA 5% in PBS buffer	A centrifuge step is required to extract the human serum	[20]
Point-of-care aptamer-tethered enzyme capture	Col	5 ng/mL–5 μg/mL 5 ng/mL	Whole blood	Syringe-based and well-based methods. Some challenges with LOD	[23]
Microfluidic aptamer-tethered enzyme capture	Col	5 ng/mL–5 μg/mL 5 ng/mL	Whole blood	Complex microfluidic chamber design	[24]
Non-natural cubamer aptamer-mediated assay	Col	4 ng/mL–4 μg/mL 4 ng/mL	Human serum	Requires unusual nucleotide chemistry oligonucleotides	[43]
ELISA	Col	3.91–250 ng/mL 3.91 ng/mL	Human serum Lysed blood	Expensive and time-consuming assay	[22]
Magnetic microparticle-quantum dot-AuNP	Col	6.6–660 pg/mL 6.6 pg/mL	Buffer	Very low LOD but relatively narrow DR Not tested in real or simulated serum and/or blood sample	[21]
Microfluidic microplate	CL	1–100 ng/mL 1 ng/mL	Human serum	A centrifuge step is required to extract the human serum	[44]
DNA-scaffolded silver nanoclusters	Fl	7.4 ng/mL–450 μg/mL 7.4 ng/mL	PBS	Centrifuge and dilution steps are required to carry out the measurement in serum	[28]
Integrated magnetic bead-quantum dot	Fl	0.1–10 ng/mL 0.1 ng/mL	Human serum	A centrifuge step is required to extract the human serum	[29]
Immuno-PCR	Fl	0.02–200 p/μL (2.4 pg/mL–24 ng/mL) 0.02 p/μL (2.4 pg/mL)	Buffer	Time-consuming assay Not feasible in complex matrix	[16]
Honeycomb array of AuNPs	Fl	0.35 pg/mL–35 ng/mL 0.6 pg/mL	Whole blood	Relatively long fabrication Conceived for ultrasensitive assay	[12]
Randomly positioned AuNPs	Fl	0.3 ng/mL–300 μg/mL 0.3 ng/mL	Whole blood	DR spans six decades The test requires only a blood dilution LOD can be reduced by using human serum	Present work

*EC* electrochemical, *O-EC* opto-electrochemical, *Col* colorimetric, *CL* chemiluminescence, *Fl* fluorescence, *p* parasite

## Conclusions

The 2D array of AuNPs herein described was implemented as a fluorescence enhancer in an apta-immunosensor for detecting malaria marker in human blood. Extremely high specificity, competitive LOD—better than that achieved by ELISA—and scalable fabrication are the main strengths of the proposed device. In addition, the gold surface biofunctionalization carried out by PIT contributes to increase the detection efficiency as well as the long-term stability. Although there are still some areas that need further optimization (e.g., a fluidic system), such a plasmonic substrate may constitute the bottom of a multiwell plates paving the way to high-throughput analysis, a feature that makes the device promising in several biological applications since the extension to other analytes can be immediately achieved by properly adapting the sandwich scheme. Moreover, it is expected that the reported LOD lying in the picomolar range may be pushed down to femtomolar level if a transparent matrix (e.g., human serum) rather than whole blood is analysed.

## Supplementary information

ESM 1 (PDF 2460 kb)

## Data Availability

The data that support the findings of this study are available from the authors on reasonable request.

## Code availability

The codes used to analyse the data are available from the authors on reasonable request.
