# Supplementary material for: Randomly positioned gold nanoparticles as fluorescence enhancers in apta-immunosensor for malaria test
Source: Mikrochim Acta. 2021 Feb 16;188(3):88. doi: 10.1007/s00604-021-04746-9 (PMC7886758; doi:10.1007/s00604-021-04746-9)
Supplement: Supplementary file 1 — (PDF 2460 kb) [file 604_2021_4746_MOESM1_ESM.pdf]

Electronic Supporting Material

Randomly positioned gold nanoparticles as fluorescence enhancers in apta-immunosensor for malaria test

Antonio Minopoli[1,2], Bartolomeo Della Ventura[2], Raffaele Campanile[2], Julian A. Tanner[3], Andreas Offenhäusser[1], Dirk Mayer[1,*], Raffaele Velotta[2,*]

[1]Institute of Biological Information Processing (IBI-3), Bioelectronics, Forschungszentrum Jülich, 52425 Jülich, Germany
[2]Department of Physics "E. Pancini", University Federico II, Via Cintia 26, 80126 Naples, Italy
[3]School of Biomedical Sciences, University of Hong Kong

*Corresponding authors: rvelotta@unina.it, dirk.mayer@fz-juelich.de

Table of content

S1. Reagents and materials.

S2. Synthesis of gold nanoparticle.

S3. Fabrication of randomly positioned AuNP array.

S4. Processing of scanning electron micrographs.

S5. Numerical simulation.

S6. Substrate biofunctionalization through photochemical immobilization technique.

S7. Microfluidic system.

S8. Acquisition of fluorescence images.

S9. Processing of fluorescence images.

S10. Protocol for detecting *Pf*LDH in blood.

S11. Optical and morphological characterization of citrate-stabilized AuNPs.

S12. Morphological characterization of the substrate.

S13. Functionalization study.

S14. Fabrication and functionalization reproducibility

S15. Study of Ab-analyte and Apt*-analyte binding processes.

S16. Kinetic curves.

S17. Fluorescence images at different analyte concentration.

S18. Fluorescence spot analysis.

S19. 5-FAM drop fluorescence intensity.

S1. Reagents and materials.

Gold(III) chloride trihydrate ($HAuCl_4 \cdot 3H_20$), sodium citrate dihydrate and (3-Aminopropyl)triethoxysilane (APTES) were purchased from Sigma-Aldrich; acetone (≥ 99.0%), 2-propanol (≥ 99.5%) and ethanol (≥ 99.5%) were purchased from Merck Millipore; bovine serum albumin (BSA) (fraction V IgG free, fatty acid poor) was obtained from Gibco. Ultrapure deionized water used for all aqueous solutions was dispensed by Milli-Q system (18.2 MΩ cm resistivity). 10 mM phosphate-buffered saline (PBS) (NaCl 10 mM, NaH_2PO_4 10 mM, Na_2HPO_4 10 mM, $MgCl_2$ 1 mM, pH 7.1) and 25 mM Tris-HCl buffer (NaCl 100 mM, imidazole 20 mM, Tris 25 mM, HCl 25 mM, pH 7.5) were prepared by dissolving the reagents (purchased from Sigma-Aldrich) in ultrapure water. Pan malaria antibody (monoclonal anti-*P*LDH antibody clone 19g7) was produced by Vista Laboratory Services (Langley, USA). Recombinant *Plasmodium falciparum* lactate dehydrogenase (*Pf*LDH) were obtained from bacterial expression [1]. Malaria 2008s aptamer labelled with 5-carboxyfluorescein tag (5’-5-FAM-CTG GGC GGT AGA ACC ATA GTG ACC CAG CCG TCT AC-3’) [1] was synthesized by Friz Biochem GmbH (Neuried, Germany). Superslip cover slips (borosilicate glass, thickness 0.13-0.17 mm) were purchased from Thermo Fisher Scientific.

S2. Synthesis of gold nanoparticle.

Gold nanoparticles (AuNPs) were synthesized by chemical reduction of gold(III) chloride trihydrate with sodium citrate (Fig. S1) [2]. Particularly, 3.4 mg of $HAuCl_4 \cdot 3H_20$ was dissolved into 50 mL of ultrapure water and the resulting solution was warmed at 150 °C under vigorous stirring. A volume of 6 mL sodium citrate (34 mM) was added during the boiling to lead particle nucleation. A volume of 4.2 mL solvated $HAuCl_4 \cdot 3H_20$ (17 mM) was spiked into the solution after 2 min in order to induce the particle growth. Within few minutes, the colour of the solution went from yellowish to black and then to bright red. The colloidal AuNP solution was cooled down at room temperature for 2 h under vigorous stirring and dark condition.

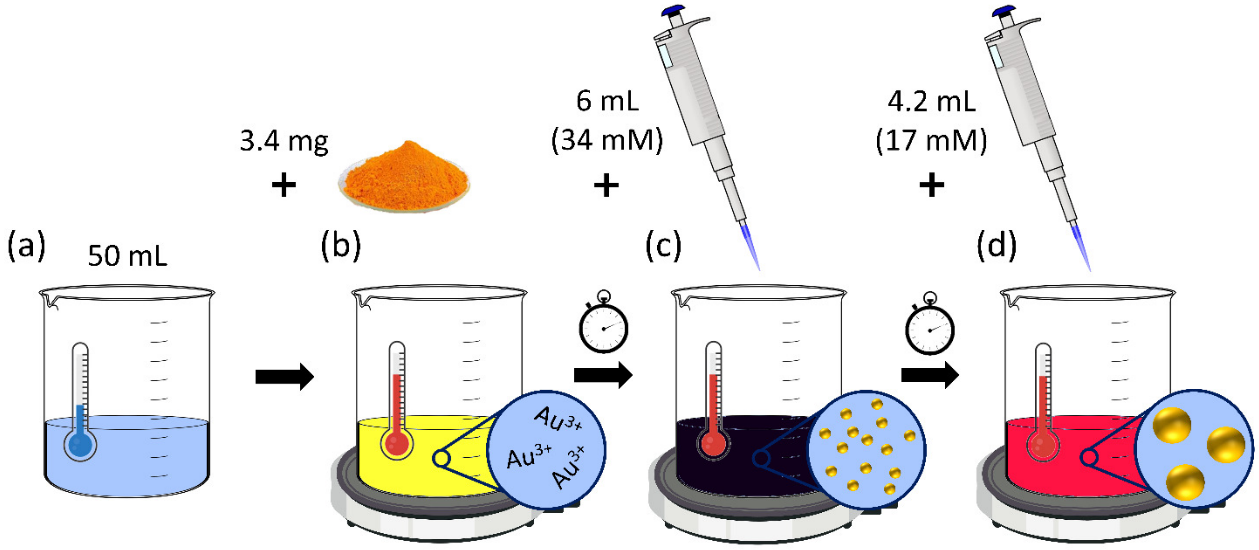

Fig. S1. Schematic representation of AuNP synthesis through chemical reduction of $HAuCl_4$. (a) Ultrapure water as solvent prevents salt-induced aggregation during the AuNP synthesis. (b) Dissolution of the gold precursor and heating of the solution under vigorous stirring. (c) Addition of sodium citrate during boiling induces the formation of gold seeds. The colour of the solution appears blackish. (d) Addition of solvated $HAuCl_4$ promotes the particle nucleation. The colour of the solution moves to pink-red due to the gold growth.

S3. Fabrication of randomly positioned AuNP array.

Silanization process by (3-Aminopropyl)triethoxysilane (APTES) was employed to chemically modify the substrate surface – consisting of 10 × 8 mm^2 glass coverslips – allowing the randomly immobilization of citrate-stabilized AuNPs by simple adsorption via electrostatic interactions [3–6]. Particle density can be easily tuned by changing the citrate-stabilized AuNP concentration, salt concentration, and the incubation time [4].

The procedure included five steps that are schematically shown in Fig. S2. (a) The coverslips were cleaned by sonication for 5 min in pure acetone, 2-propanol and pure ethanol sequentially, and extensively rinsed by ultrapure water. (b) The glass surface was activated by oxygen plasma treatment (1.4 mbar pressure, 200 W power, 2 min) leading to its oxidation and the formation of silanol bonds, which served as bonding sites for silane molecules. The silanols provided the glass with hydroxyl surface functionality that gave rise to a high negative surface charge. (c) The activated substrates were modified with APTES by controlled low-pressure evaporation inside a vacuum desiccation chamber (argon gas, 5 mbar pressure, 1 h) that provided the glass surface with a monolayer of positively charged amino-terminated silanes. Silane multilayers could be easily formed during the silanization procedure causing the subsequent AuNP aggregation. In order to avoid the silane polymerization, the abrupt desiccation of APTES onto glass surface had to be avoided by smoothly changing the pressure level into the desiccation chamber. (d) The silanized substrates were incubated with 1 mL citrate-stabilized AuNPs (OD ~1.0) for 4 h at room temperature and dark condition. The electrostatic interaction between the negatively charged citrate shell of AuNPs and the positively charged amino groups of silanes warranted a strong immobilization of citrate-AuNPs onto glass coverslips. (e) A low-pressure oxygen plasma treatment (0.8 mbar pressure, 200 W power, 30 min) etched the free citrate ligands and silane layer leaving the bare AuNPs strongly anchored to the substrate.

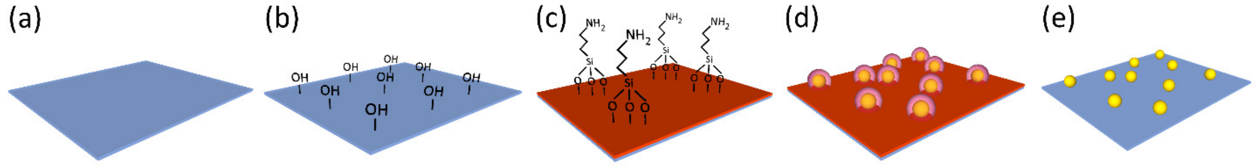

Fig. S2. Fabrication process of 2D AuNP array by nanolithography based on silane surface modification. (a) Substrate cleaning. (b) Surface activation by oxygen plasma. (c) Surface silanization. (d) Citrate-AuNP immobilization. (e) Citrate and silane layer etching.

S4. Processing of scanning electron micrographs.

The substrate was morphologically characterized by Zeiss LEO 1550VP field emission scanning electron microscope (FESEM) with a nominal resolution of 1 nm at 20 kV acceleration voltage. The recorded scanning electron micrographs were processed by ImageJ software to retrieve information about the shape and size of nanoparticles and the center-to-center distance. Firstly, raw images (Fig. S3a shows an example of micrograph acquired at high magnification) were thresholded thereby isolating nanoparticles from the background (Fig. S3b). Then, adjacent objects were separated by "Watershed" tool implemented in ImageJ (Fig. S3c). Eventually, perimeter p, area S, centroid coordinates and shape descriptors (circularity $C = 4\pi S/p^2$ and aspect ratio AR) were measured by decomposing each object in outline and inner region through "Analyze Particles" tool implemented in ImageJ (Fig. S3d).

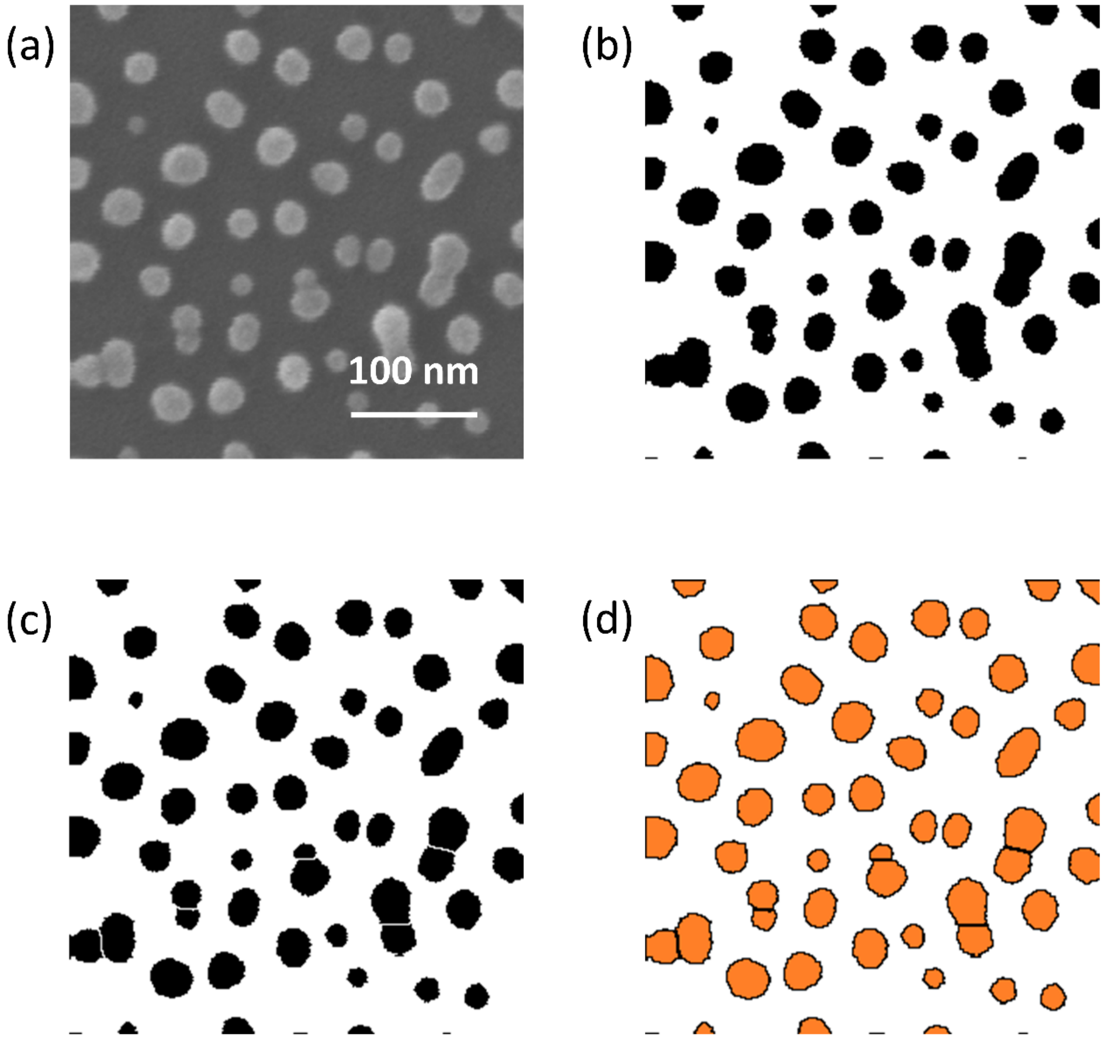

Fig. S3. SEM micrograph analysis. (a) Example of raw top view SEM micrograph at high magnification. Corresponding (b) thresholded and (c) segmented image. (d) Processed image in which the objects are decomposed in outlines (black line) and inner region (orange filling).

S5. Numerical simulation.

The optical response of the 2D AuNP array was simulated by using the finite-difference time-domain (FDTD) method implemented in "FDTD solutions" tool of Lumerical software that solves Maxwell's equations within a Mie problem-like workspace. Such a workspace, consisting of light source, nanostructure, photodetector, and boundary conditions (BCs), was discretized over a mesh (1 nm spatial resolution) so that Maxwell's equation could be numerically solved in the time domain by evaluating the evolution of the electromagnetic field in each sampled volume. An illustration of the simulation workspace is shown in Fig. S4. Linearly *x*-polarized plane wave traveling along *z* direction was set to investigate the system, whereas a photodetector was positioned on the opposite side of the workspace to measure the extinction spectrum of the substrate. The latter was modelled by importing the real morphology provided by scanning electron micrographs, in which homogeneous gold nanoparticle were placed onto a thick dielectric layer of silicon dioxide. Symmetric/anti-symmetric BCs were set along *x* and *y* direction to reduce the simulation time by a factor 8 without worsening the result accuracy, whereas perfect matched layer BCs along *z* direction warrant perfect absorption of the electromagnetic radiation both propagating beyond the photodetector and backscattered through the light source.

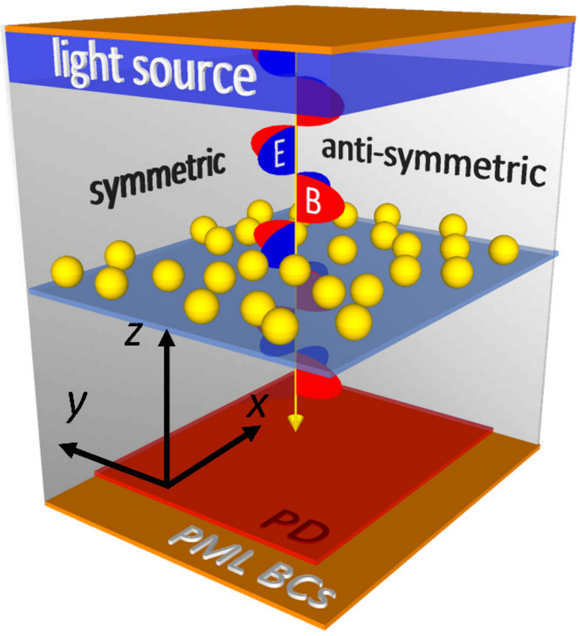

Fig. S4. Sketch of the simulation workspace consisting of plane wave source, plasmonic nanostructure, dielectric substrate (SiO_2 glass), photodetector (PD) and appropriate boundary conditions.

S6. Substrate biofunctionalization through photochemical immobilization technique.

The UV irradiation of Abs produces the selective photoreduction of the disulfide bridge (S-S) in cysteine-cysteine/tryptophan (Cys-Cys/Trp) triads giving rise the breakage of Cys-Cys bonds and the formation of free thiol groups (-SH) (Fig. S5a). Such thiol-terminated ends are localized in both Ab Fab fragments and have high chemical reactivity with noble metals. Functionalization by PIT warrants both the covalent tether of Abs onto the AuNP surface and the control over their orientation so to achieve the exposure of one of the two antigen binding sites to the surrounding environment (Fig. S5b) [7].

The UV source consisted of two U-shaped low-pressure mercury lamps (power of single lamp 6 W at 254 nm) that wrapped the quartz cuvette containing the Ab aqueous solution delivering approximately 0.3 W/cm^2 (Fig. S5c). Such an irradiation intensity was strong enough to warrant the selective thiol group production in both Ab Fab fragments while prevented significant direct photolysis of S-S bonds that poorly absorb at 254 nm [8].

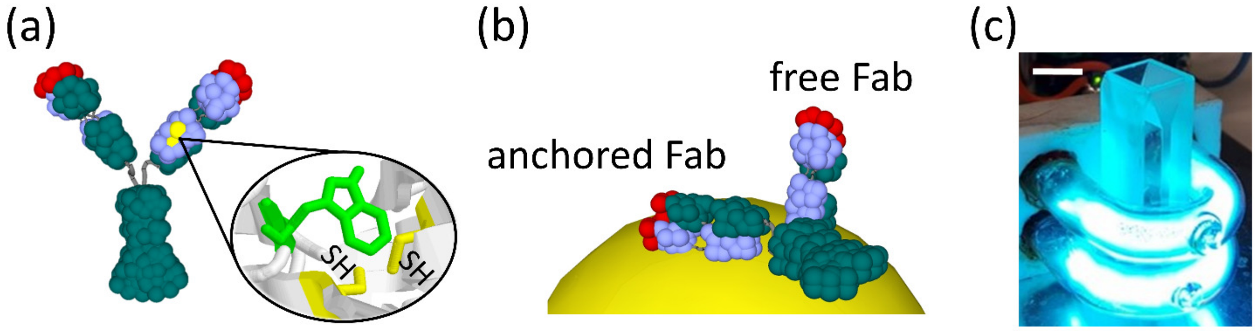

Fig. S5. Gold nanoparticle biofunctionalization through PIT. (a) UV irradiation of Abs leads to the production of four thiol groups (-SH) (two of them are not visible in the illustration). (b) Abs tether onto the nanoparticle gold surface exposing one of their binding sites to the surrounding environment. (c) Low pressure mercury U-shaped UV lamps (Trylight UV-lamp).

S7. Microfluidic system.

The functionalization was carried out by using a microfluidic circuit consisting of a cell containing the substrate, a 2 mL syringe, and Tygon tubes with a diameter of 1 mm (for both the input and output channel) designed for biological samples (Fig. S6). The volume of the solution in contact with the substrate was approximately 30 μL, whereas the total volume flowing into the circuit was ~200 μL. The syringe was used to repeatedly draw 250 μL of the fresh aqueous solution containing the irradiated Abs (4 draws separated by a time interval of 3 min). Then, the substrate was copiously rinsed by ultrapure water to remove unbound Abs. Aqueous solution containing BSA proteins (50 μg/mL) was used to block free AuNP surface from any nonspecific adsorption (4 draws of 250 μL separated by a time interval of 1 min).

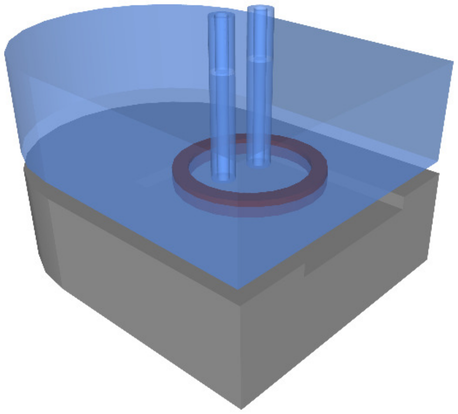

Fig. S6. Sketch of the fluidic cell used to promote an effective interaction of the irradiated Abs contained into the aqueous solution with the nanostructured substrate.

S8. Acquisition of fluorescence images.

Fluorescence images were recorded by Zeiss Axio Observer Z1 inverted phase contrast fluorescence microscope. The fluorescence microscope was equipped by Zeiss Colibri.2 LED light source (module 470 nm), Zeiss Plan-Apochromat 10x/0.45 Ph1 M27 (FWD = 2.1 mm) objective, 38 HE filter (excitation 450-490 nm/emission 500-550nm) and pco.edge 5.5 sCMOS photodetector (scaling 0.650 µm × 0.650 µm, image size 2560 × 2160 pixels, scaled image size 1.66 mm × 1.40 mm, 16-bit dynamic range). The camera exposure time was set to 2 s for recording every image.

S9. Processing of fluorescence images.

Image processing and analysis were performed by ImageJ software. Raw fluorescence images exhibited smooth continuous background that was removed by the so-called "rolling ball" algorithm. Such an algorithm locally measured the background by averaging it over a ball around each pixel. The resulting mean values were subtracted from the raw image so that the background was flattened (see Fig. S7). A sphere of 20 pixels diameter represented the best compromise to properly include the largest objects that were not part of the background and to warrant a flat and homogeneous background. Aiming at measuring the whole fluorescence intensity, the images were thresholded at a value slightly higher than the flattened background – so that the resulting background value was zero – and the signals arising from the fluorescence spots were summed.

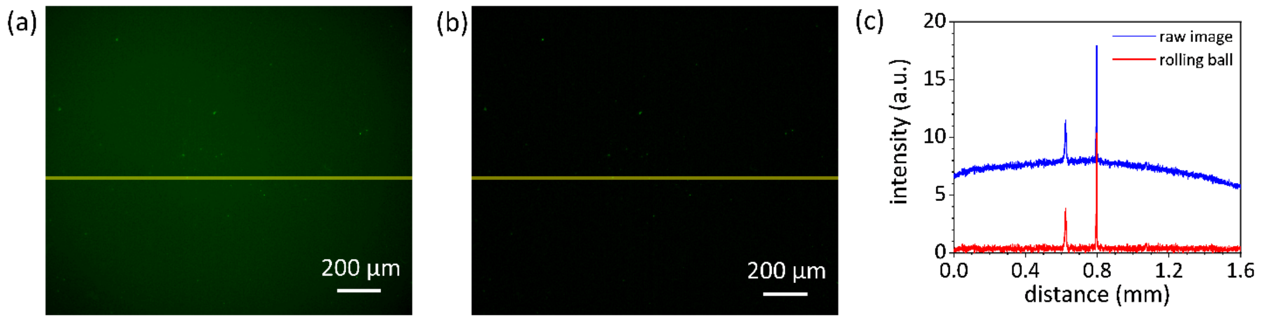

Fig. S7. Fluorescence image processing. (a) Example of raw fluorescence image recorded at low *Pf*LDH concentration. The significantly high non-flat background is removed by the "rolling ball" algorithm. (b) The resulting image exhibits a flat background, from which the fluorescence spots are well distinguishable. (c) Intensity profile evaluated along the yellow line highlighted in panels (a) and (b).

S10. Protocol for detecting *Pf*LDH in blood.

Once the device is ready to be used, the following steps allow one to measure the *Pf*LDH concentration in human blood:

1) Dilute 10 μL of patient's blood in 990 μL of 25 mM Tris buffer (see Table S1 for the preparation of Tris buffer).
2) Incubate anti-*P*LDH-functionalized substrate with 1 mL blood sample by gently shaking the bowl for 50 min.
3) Rinse by sequentially dipping the substrate in 25 mM Tris buffer and ultrapure water (three times).
4) Incubate the substrate with 10 mM PBS solution containing 0.1 μM 5-FAM-labelled malaria aptamers by gently shaking the bowl for 30 min in dark condition (see Table S2 for the preparation of aptamer-spiked PBS).
5) Rinse by sequentially dipping the substrate in 10 mM PBS and ultrapure water (three times).
6) Blow-dry the substrate with nitrogen.
7) Acquire fluorescence images of the substrate at 520 nm (excitation wavelength 490 nm) (see section S8 for more details).
8) Process the raw image and analyse (see section S9 for more details).

Table. S1. Tris buffer solution was prepared by dissolving the reagents into 500 mL of ultrapure water under vigorous stirring at room temperature. The buffer pH was adjusted to 7.5 by adding 750 µL of HCl 37%.

**Reagent**	**State**	**Added amount**	**Final concentration**
NaCl	powder	2.922 g	100 mM
Imidazole	powder	0.681 g	20 mM
Tris base	powder	1.515 g	25 mM
HCl 37%	liquid	397 µL	-

Table. S2. PBS solution was prepared by dissolving the reagents into 100 mL of ultrapure water under vigorous stirring at room temperature. The buffer pH was adjusted to 7.1 by adding 250 µL of NaOH (4 M). A volume of 1.8 µL of 5-FAM-labelled malaria aptamers (56.9 µM) was spiked into 1 mL of 10 mM PBS to obtain the final concentration of 0.1 µM.

**Reagent**	**State**	**Added amount**	**Final concentration**
NaCl	powder	585 mg	100 mM
NaH_2PO_4	powder	140 mg	10 mM
Na_2HPO_4	powder	140 mg	10 mM
$MgCl_2$	powder	9.5 mg	1 mM

S11. Optical and morphological characterization of citrate-stabilized AuNPs.

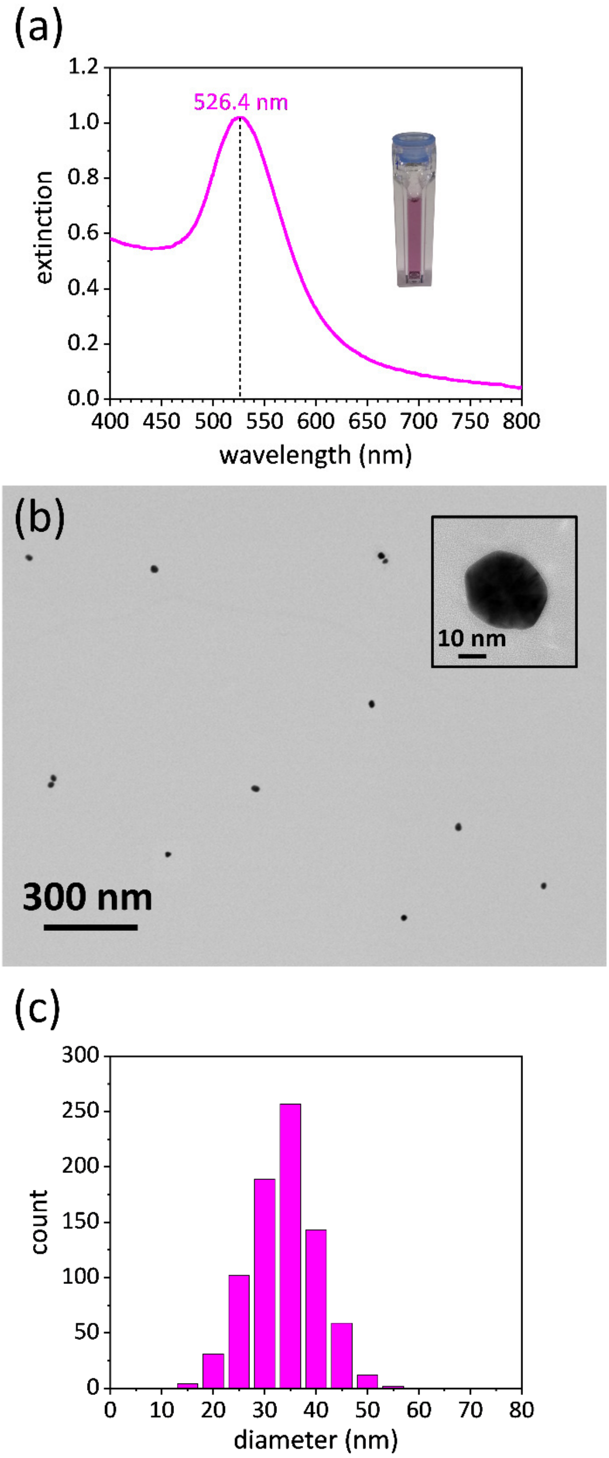

Fig. S8. Optical and morphological characterization of citrate-stabilized AuNPs. (a) Extinction spectrum of the colloidal solution containing citrate-stabilized AuNPs (corresponding photograph in the inset). (b) STEM micrograph of bare AuNPs at low magnification, the inset shows a single nanoparticle at high magnification. (c) Bare AuNP diameter distribution.

S12. Morphological characterization of the substrate.

Nanoparticle diameter was estimated as $D = 2\sqrt{S/\pi}$ while interparticle distance by computing the distance among each centroid and its nearest neighbours. Size distribution is peaked at approximately 31 nm with a standard deviation of 12 nm (Fig. S9a) pointing out no morphological changes were induced during the substrate fabrication process. Right tail of the histogram is due to AuNP clusters as a byproduct of silane-induced aggregation. Fig. S9b shows the histogram of the centre-to-centre distances whose distribution turns out to be quite broad with 67 nm mean value and 19 nm standard deviation as a result of the randomness of the nanoparticle immobilization onto the substrate. Fig. S9c and S9d shows the distributions concerning the aspect ratio AR and circularity C, respectively, of the immobilized AuNPs. The mean values $\overline{AR} = 1.4 \pm 0.4$ and $\bar{C} = 0.90 \pm 0.14$ convey high regularity in nanoparticle roundness held at macroscopic level.

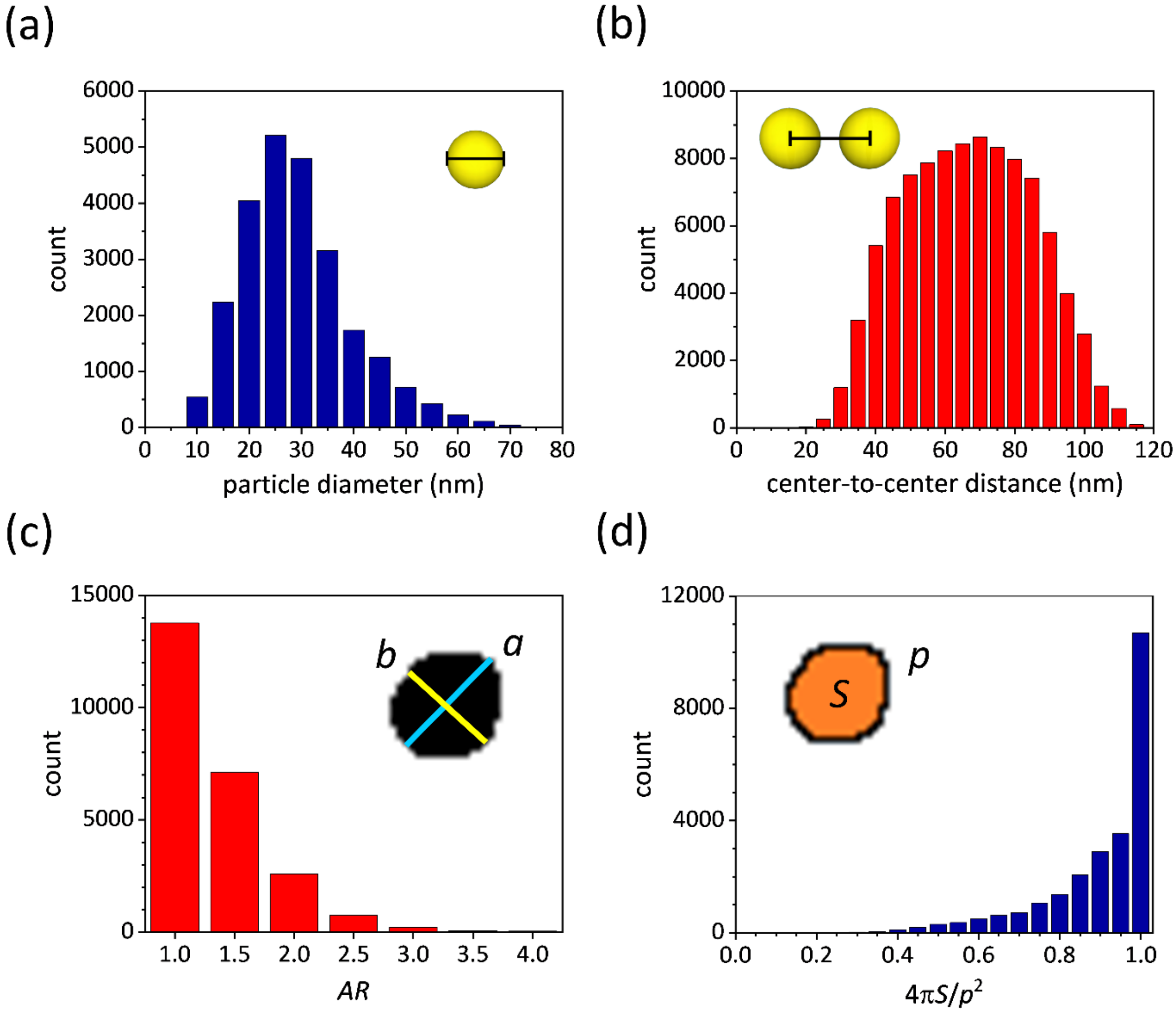

Fig. S9. Morphological characterization of the substrate. (a) Nanoparticle diameter distribution. (b) Center-to center distance distribution. (c) Histogram of AuNP aspect ratio, the highlighted lines in the inset represent the major (light blue) and minor (yellow) axis of the examined object. (d) Histogram of AuNP circularity, the inset shows an example of a nanosphere decomposed in perimeter (black line) and area (orange filling).

S13. Functionalization study.

The coverage of AuNPs with a dielectric protein layer leads to a red-shift of the LSPR wavelength up to 4 nm at 50 µg/mL concentration of anti-*P*LDH. For larger concentrations no change in plasmon resonance was observed due to the saturation of the free gold surface (Fig. S10a). By considering the close-packing arrangement offered by PIT, the number of Abs that can be anchored onto a 30 nm diameter nanosphere is approximately 20 [9]. The excellent covering of the AuNP surface by Abs is also evident by the lack of any significant optical change in the substrate extinction spectrum after the blocking step (blue continuous line in Fig. S10b).

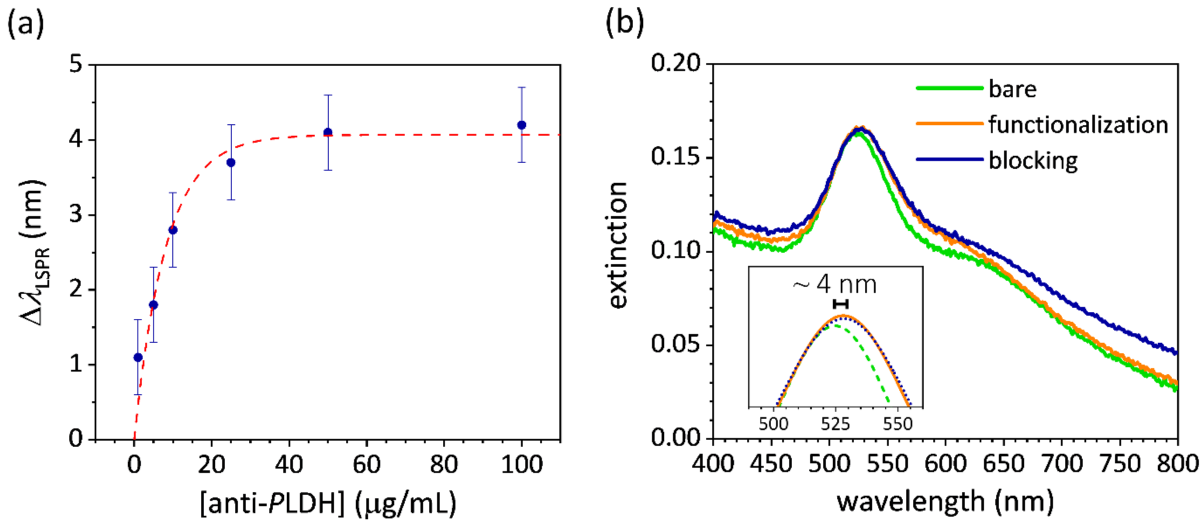

Fig. S10. (a) Experimental extinction spectrum of bare substrate (green continuous line), substrate functionalized through PIT (orange continuous line), and substrate after blocking (blue continuous line). The inset shows the Gaussian fits used for evaluating the LSPR wavelength. (b) Shift of the LSPR wavelength as a function of anti-*P*LDH concentration. The LSPR peak red-shifts as the anti-*P*LDH concentration increased up to 50 µg/mL that corresponded to a maximum red-shift of 4 nm. The data are well fitted by exponential curve (dashed red line).

S14. Fabrication and functionalization reproducibility.

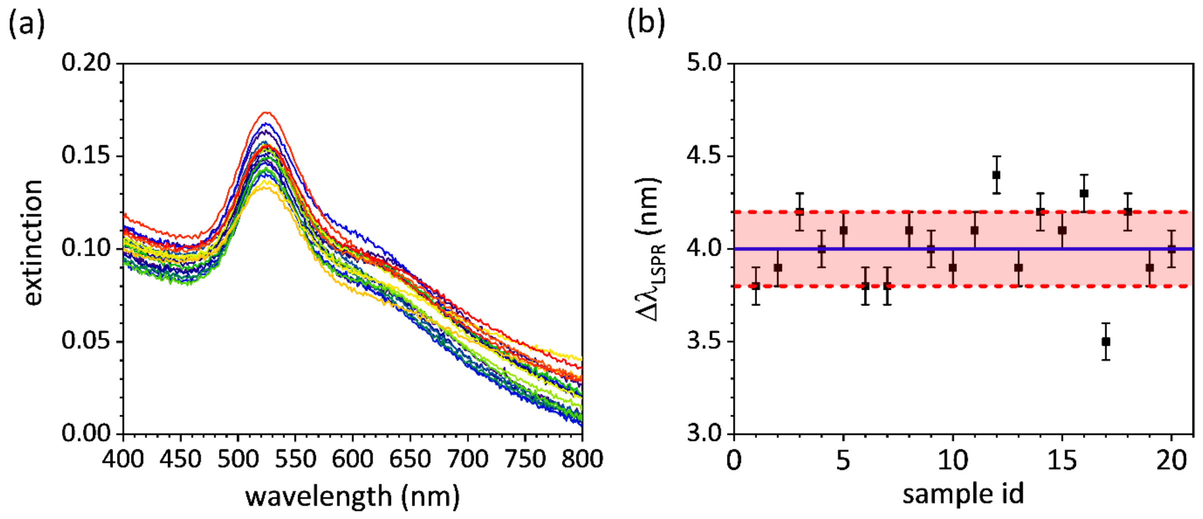

Fig. S11. (a) Experimental extinction spectrum of twenty bare substrates. The simplicity in the fabrication warrants high reproducibility in the substrate optical response. (b) LSPR shift measured for twenty substrates after anti-*P*LDH functionalization. The blue line is the mean value of the measurements, whereas the red band highlights the $\pm\sigma$ interval. Only three measurements out of twenty fall outside the 67% confidence interval confirming a good repeatability of the functionalization process.

S15. Study of Ab-analyte and Apt*-analyte binding processes.

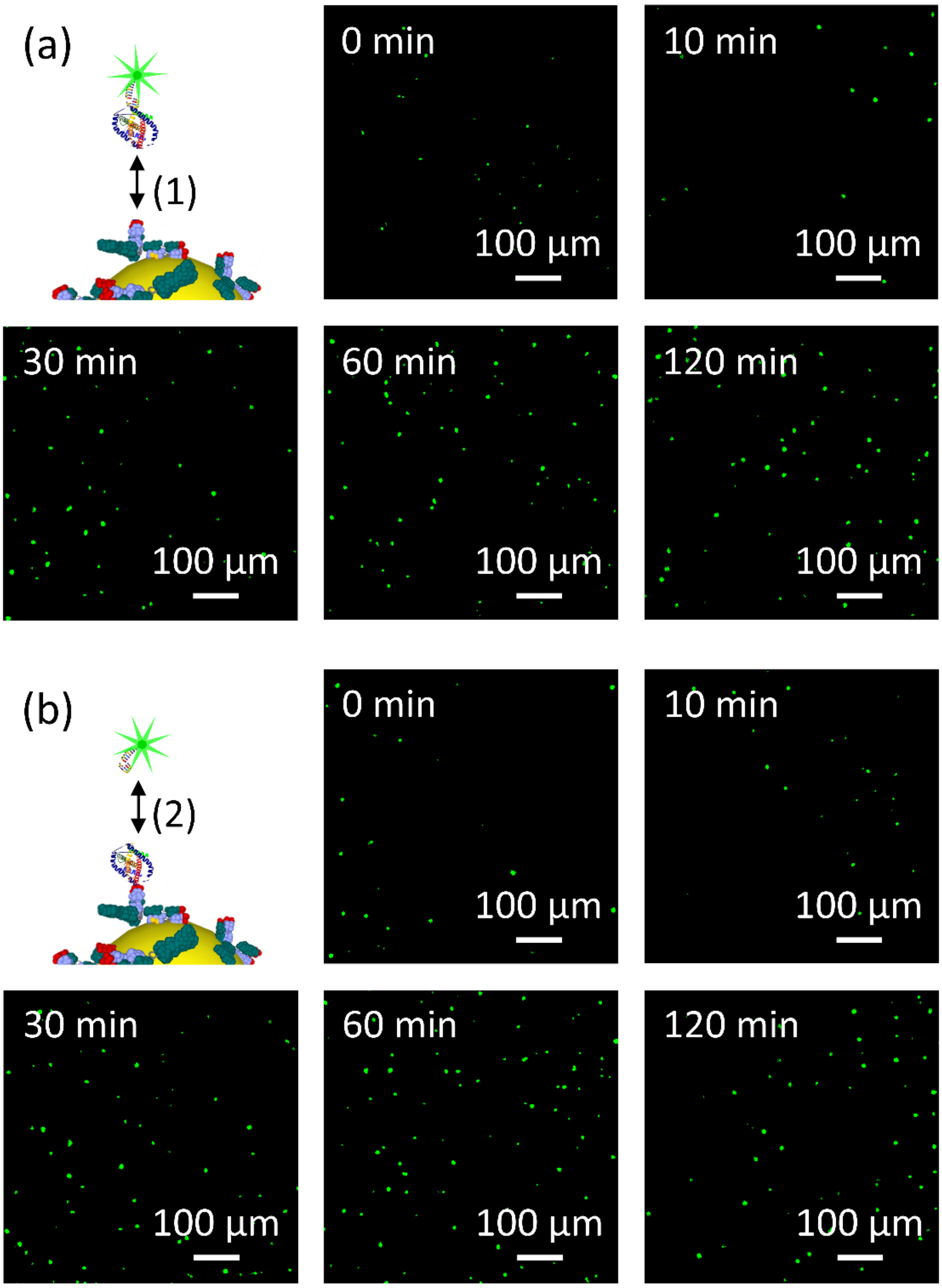

Fig. S12. Example of fluorescence images recorded at different incubation time for both (a) Ab-analyte and (b) Apt*-analyte binding processes.

S16. Kinetic curves.

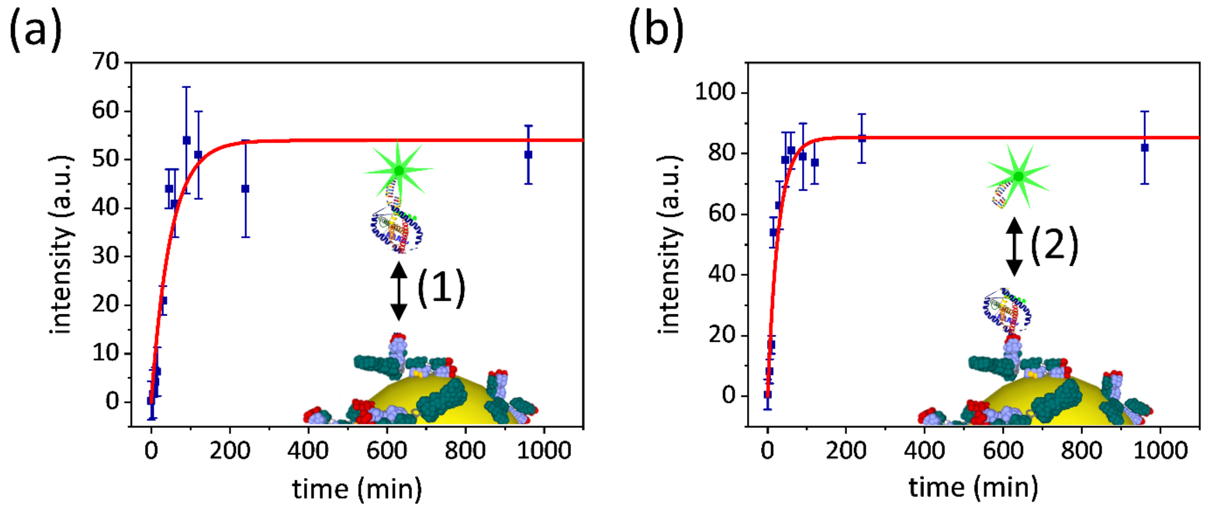

Fig. S13. Kinetic curves related to (a) Ab-analyte and (b) Apt*-analyte binding dynamics. The data are well fitted by exponential curves (red continuous lines).

S17. Fluorescence images at different analyte concentration.

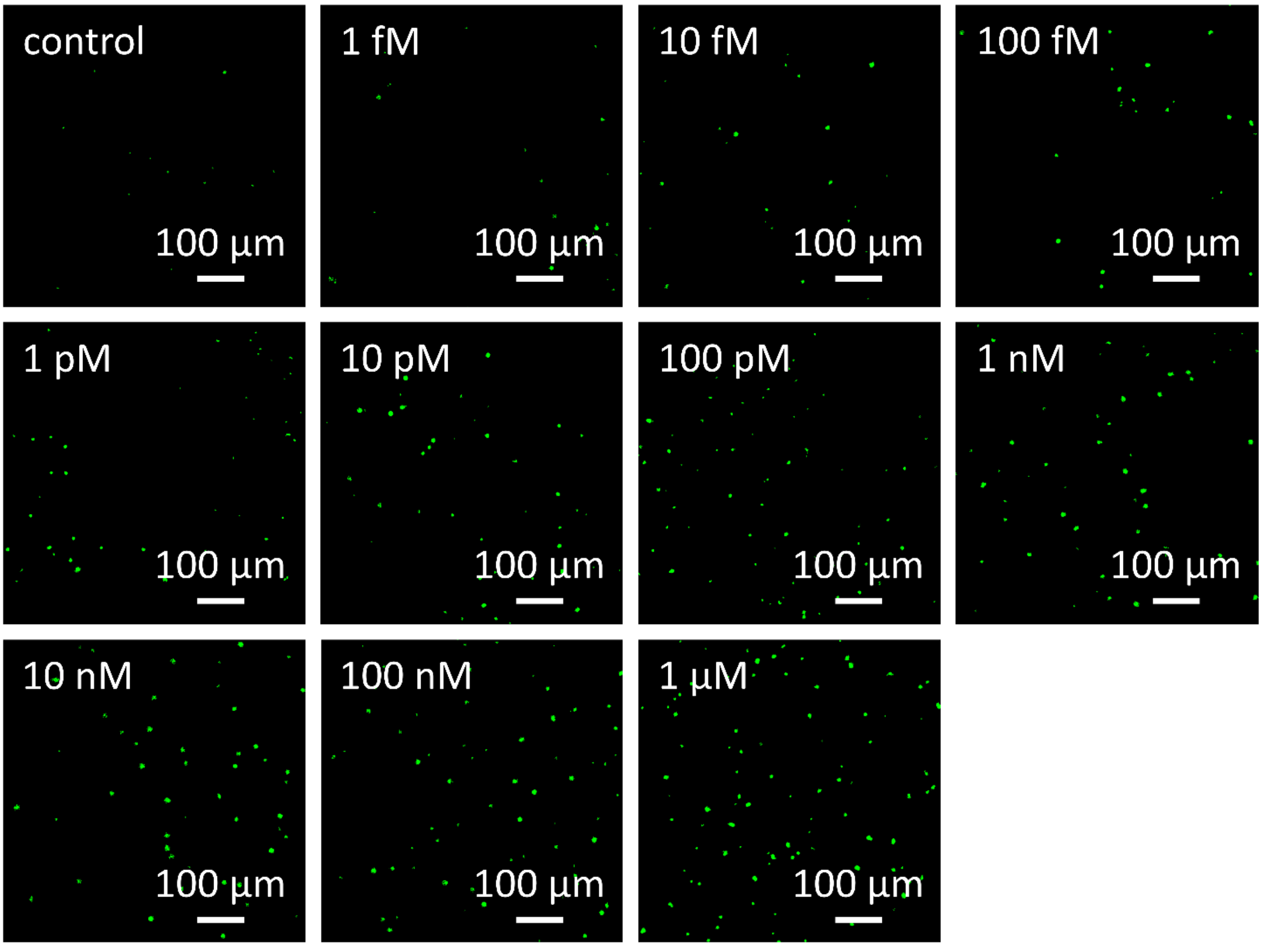

Fig. S14. Example of fluorescence images recorded at different *Pf*LDH concentrations spiked into uninfected whole blood.

S18. Fluorescence spot analysis.

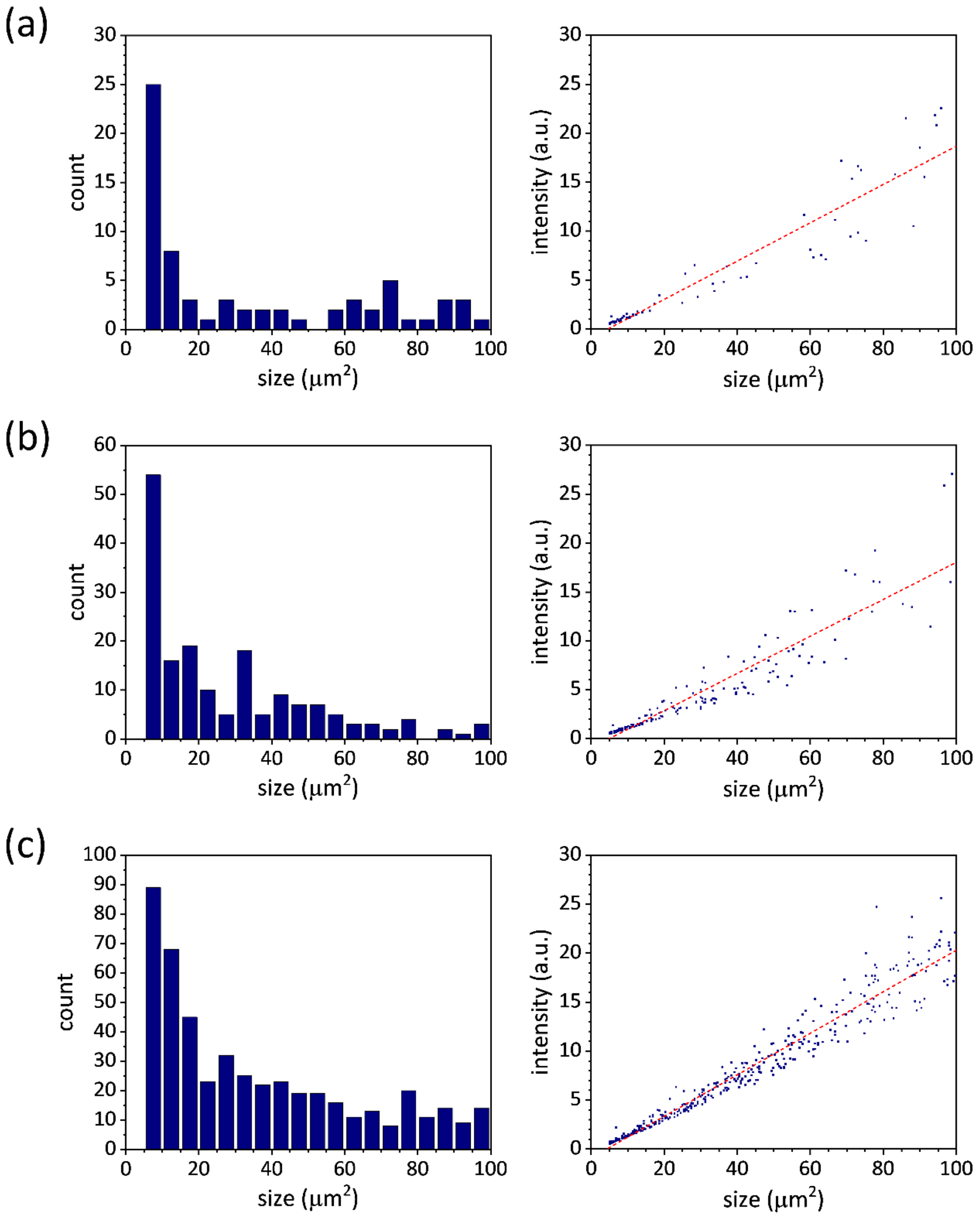

Fig. S15. Fluorescence spot analysis. Spot size distribution and correlation intensity vs spot area at (a) 1 pM, (b) 1 nM and (c) 1 μM *Pf*LDH concentration spiked into uninfected whole blood show that the mean spot area does not significantly depend on the analyte concentration, whereas the fluorescence intensity of the emitter is strongly related to the number of pixels that collect the signal (Pearson's r = 0.95).

S19. 5-FAM drop fluorescence intensity.

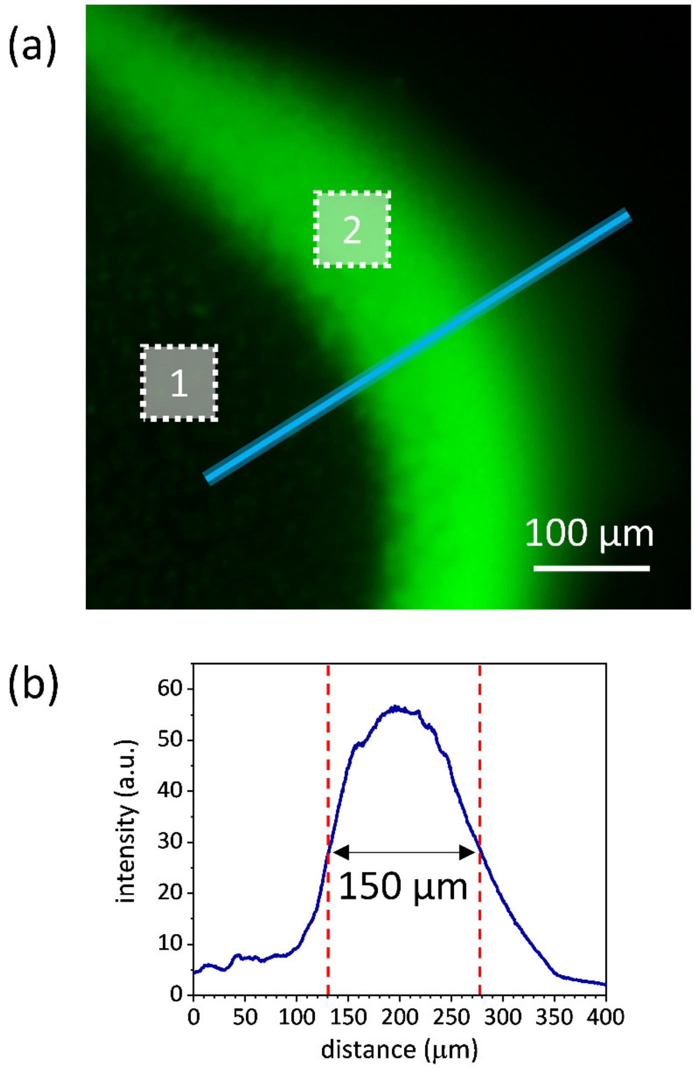

Fig. S16. 5-FAM drop fluorescence intensity. (a) Example of fluorescence image recorded in close proximity of the drop edge. The whole intensity of the 5-FAM drop was measured by sampling the drop area in one hundred regions of interest of size 350 × 350 µm^2 in the inner region (white square 1, not in scale) and 70 × 70 µm^2 on the annulus (white square 2). (b) Ring thickness measured as FWHM of the intensity profile evaluated along the light blue line highlighted in panel (a).

References

1. Cheung Y-W, Kwok J, Law AWL, et al (2013) Structural basis for discriminatory recognition of Plasmodium lactate dehydrogenase by a DNA aptamer. Proc Natl Acad Sci 110:15967–15972. https://doi.org/10.1073/pnas.1309538110

2. Pollitt MJ, Buckton G, Piper R, Brocchini S (2015) Measuring antibody coatings on gold nanoparticles by optical spectroscopy. RSC Adv 5:24521–24527. https://doi.org/10.1039/C4RA15661G

3. Greben K, Li P, Mayer D, et al (2015) Immobilization and Surface Functionalization of Gold Nanoparticles Monitored via Streaming Current/Potential Measurements. J Phys Chem B 119:5988–5994. https://doi.org/10.1021/acs.jpcb.5b02615

4. Gilles S, Kaulen C, Pabst M, et al (2011) Patterned self-assembly of gold nanoparticles on chemical templates fabricated by soft UV nanoimprint lithography. Nanotechnology 22:295301. https://doi.org/10.1088/0957-4484/22/29/295301

5. Markov A, Greben K, Mayer D, et al (2016) In Situ Analysis of the Growth and Dielectric Properties of Organic Self-Assembled Monolayers: A Way to Tailor Organic Layers for Electronic Applications. ACS Appl Mater Interfaces 8:16451–16456. https://doi.org/10.1021/acsami.6b04021

6. Yuan X, Wolf N, Mayer D, et al (2019) Vapor-Phase Deposition and Electronic Characterization of 3-Aminopropyltriethoxysilane Self-Assembled Monolayers on Silicon Dioxide. Langmuir acs.langmuir.8b03832. https://doi.org/10.1021/acs.langmuir.8b03832

7. Della Ventura B, Banchelli M, Funari R, et al (2019) Biosensor surface functionalization by a simple photochemical immobilization of antibodies: experimental characterization by mass spectrometry and surface enhanced Raman spectroscopy. Analyst 144:6871–6880. https://doi.org/10.1039/C9AN00443B

8. Neves-Petersen MT, Klitgaard S, Pascher T, et al (2009) Flash Photolysis of Cutinase: Identification and Decay Kinetics of Transient Intermediates Formed upon UV Excitation of Aromatic Residues. Biophys J 97:211–226. https://doi.org/10.1016/j.bpj.2009.01.065

9. Della Ventura B, Iannaccone M, Funari R, et al (2017) Effective antibodies immobilization and functionalized nanoparticles in a quartz-crystal microbalance-based immunosensor for the detection of parathion. PLoS One 12:e0171754. https://doi.org/10.1371/journal.pone.0171754
